# Opinions on registering trial details: a survey of academic researchers

**DOI:** 10.1186/1472-6963-8-18

**Published:** 2008-01-23

**Authors:** Martin Scherer, Sven Trelle

**Affiliations:** 1 Department of General Practice, University of Göttingen, Humboldtallee 38, 37073 Göttingen, Germany; 2 Institute of Social and Preventive Medicine (ISPM), University of Bern, Finkenhubelweg 11, 3012 Bern, Switzerland

## Abstract

**Background:**

The World Health Organization (WHO) has established a set of items related to study design and administrative information that should build the minimum set of data in a study register. A more comprehensive data set for registration is currently developed by the Ottawa Group. Since nothing is known about the attitudes of academic researchers towards prospective study registration, we surveyed academic researchers about their opinion regarding the registration of study details proposed by the WHO and the Ottawa Group.

**Methods:**

This was a web-based survey of academic researchers currently running an investigator-initiated clinical study which is registered with clinicaltrials.gov. In July 2006 we contacted 1299 principal investigators of clinical studies by e-mail explaining the purpose of the survey and a link to access a 52-item questionnaire based on the proposed minimum data set by the Ottawa Group. Two reminder e-mails were sent each two weeks apart. Association between willingness to disclose study details and study phase was assessed using the chi-squared test for trend. To explore the potential influence of non-response bias we used logistic regression to assess associations between factors associated with non-response and the willingness to register study details.

**Results:**

Overall response was low as only 282/1299 (22%) principal investigators participated in the survey. Disclosing study documents, in particular the study protocol and financial agreements, was found to be most problematic with only 31% of respondents willing to disclose these publicly. Consequently, only 34/282 (12%) agreed to disclose all details proposed by the Ottawa Group. Logistic regression indicated no association between characteristics of non-responders and willingness to disclose details.

**Conclusion:**

Principal investigators of non-industry sponsored studies are reluctant to disclose all data items proposed by the Ottawa Group. Disclosing the study protocol and financial agreements was found to be most problematic. Future discussions on trial registration should not only focus on industry but also on academic researchers.

## Background

Since 2005 the International Committee of Medical Journal Editors (ICMJE) has required prospective registration of clinical studies in order to be considered for publication in one of their journals [1]. Moreover, the World Health Organization (WHO) has established a set of items related to study design and administrative information that should form the minimum set of data in a study register [2]. Several stakeholders were consulted on this WHO Trial Registration Data Set including academic researchers, industry and patient representatives. However, several items were highly controversial and industry representatives demanded that some items should not be disclosed during study conduct [3]. Industry representatives argued that some trial information is sensitive for competitive reason and asked for delayed disclosure of some items e.g. the scientific title of a study, primary and secondary outcomes. However, competition is also of increasing importance in academic research [4].

In 2005 the Ottawa Group published principles of trial registration [5] and a minimum data set for registration is currently being developed [6]. This set requires even more details about a study than the WHO Trial Registration Data Set (see Table 1). However, analyses of trial registration in ClinicalTrials.gov showed heterogeneous quality and completeness of the registration entries indicating varying degrees of comfort with different levels of disclosure [7]. Since nothing is known about the attitudes of academic researchers towards prospective study registration, it is unclear if they would comply with an extension of the requested study information. We therefore surveyed academic researchers about their opinions regarding the registration of study details proposed by the Ottawa Group.

**Table 1:** Study details required by WHO and Ottawa Group

WHO Trial Registration Data Set	Ottawa Group Data Set (in addition to WHO items)
Trial registration date	Trial acronym
Ethics approval	Trial website
Funding source(s)	Lay description
Primary sponsor	Registration date
Secondary sponsor(s)	Date ethical approval
Coordinating/principle investigator	Date recruitment end
Contact person	Date end of follow-up
Coordinating center	Date trial stop
Recruitment center locations	Trial extensions
Official scientific title	Date primary analyses
Lay title	Name of Research ethics board/institutional
Date recruitment start	Review board (REB/IRB)
Recruitment status	REB trial approval number
Inclusion criteria	Rationale of the trial
Exclusion criteria	References to systematic reviews
Disease/condition	Justification of interventions
Interventions	Trial objectives
Primary endpoint	Study design
Secondary endpoints	Number of arms
Trial phase/study type	Generation of allocation sequence
Target sample size	Randomization
	Allocation concealment
	Blinding status
	Other design features
	Framework of the study
	Sample size calculation
	Planned subgroup analyses
	Planned analyses methods
	Consent forms
	Full protocol
	Contracts and financial arrangements

## Methods

### Study design

This study was a survey of academic researchers currently running investigator-initiated clinical studies which were registered with clinicaltrials.gov. It was approved by the ethics committee of the University of Göttingen, Germany.

### Study population

The basic population consisted of all recruiting or planned investigator-initiated clinical studies registered with the study register of the U.S. National Institutes of Health (clinicaltrials.gov) in May 2006 (n = 7988). Using computer generated random numbers we selected 1500 studies. Investigator-initiated studies were defined as all studies which were registered with a non-industry sponsor. In July 2006, all principal investigators with valid contact details were contacted by e-mail explaining the purpose of the survey and a link to access the questionnaire. Two reminder e-mails were sent each two weeks apart. No attempts were made to contact non-responders by phone because of difficulties to obtain sufficient contact details and resource constraints. Written informed consent was not sought in this internet based survey but informed consent of participants was implied because access to the questionnaire was restricted to persons approached by e-mail explaining the study.

### Data collection

A web-based 52-item questionnaire was developed based on the WHO Trial Registration Data Set [2] and the proposed minimum data set from the Ottawa Group [6]. Participants were asked whether they would be willing to publicly disclose specific details about their study. The questionnaire consisted of eleven main sections (Additional file 1): Contacts and funding, title and description of the study, key dates, ethical approval, background of the study, eligibility criteria, intervention(s), outcome measures, design, documents, and results. We used five response categories to indicate willingness for disclosure: yes, no, don't know/can't decide, don't want to answer, not applicable. Each question had to be answered in order to proceed with the questionnaire. Given that the questionnaire solely consisted of questions directly related to items of the two data sets mentioned above, no piloting of the questionnaire was done.

### Sample size calculation

Sample size calculation was based on confidence intervals for proportions and the following considerations: 1) a maximum width of a confidence interval for questions answerable dichotomously of 6% was considered narrow enough; 2) based on a basic population of 7988 this would require 259 responses; 3) assuming a response rate of 25% a sample size of 1036 would be needed; 4) to account for missing and invalid contact details we selected 1500 studies.

### Statistical analysis

Data were analysed in Stata 9.2 using descriptive statistics for the main analysis. Responders and non-responders were compared using the chi-squared test. Association between willingness to disclose study details and study phase was assessed using the chi-squared test for trend. To explore the potential influence of non-response bias we used logistic regression to assess associations between factors associated with non-response and the willingness to register study details. We therefore included the following factors in the regression analysis: study sponsored by the National Institutes of Health and phase II study in oncology. For ease of interpretation we recoded "yes" and "not applicable" responses as 'yes' and "no", "don't want to answer", and "don't know" as 'no'. We chose this coding procedure in order to predict willingness to disclose all study details according the Ottawa criteria. Coding "not applicable" as "no" would have had the following potential consequence: e.g. if a participant is willing to disclose the whole data-set except one item which is not applicable to his context, he would have been falsely categorized as completely reluctant (i.e. "0" in regression analysis).

## Results

**Figure 1:**
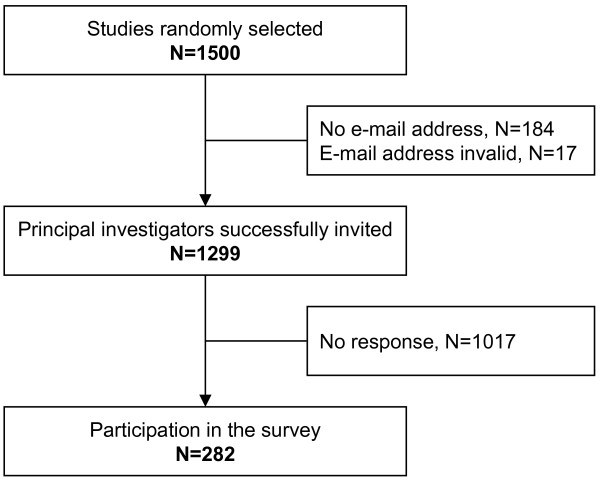
Study flow diagram.

201 out of the initial 1500 studies had no (n = 184) or no valid (n = 17) e-mail address of the principal investigator available, leaving 1299 potential participants. Studies with principal investigators with no or invalid e-mail address were more likely to be sponsored by the National Institutes of Health, to be in the field of oncology, and more likely to study a drug. Of the 1299 contacted investigators, 282 (22%) responded and these formed the population for analysis (Figure 1).

Non-responders were more likely to be principal investigators of studies sponsored by the National Institutes of Health (Table 2). Studies of non-responders were also more likely to be in the field of oncology and registered as phase II studies. Given these differences in responders and non-responders, we did not calculate confidence intervals of response distributions.

**Table 2:** Study characteristics

Variables	Total	E-mail available*	E-mail not available	p**	Responders	Non-responders	p
Recruiting status of study	1500	1299	201	0.15	282	1017	0.68
Recruiting	1375 (91.7)	1196 (92.1)	179 (89.0)		258 (91.5)	938 (92.23)	
Not yet	125 (8.3)	103 (7.9)	125 (8.3)		24 (8.5)	79 (7.8)	
Type of sponsor				<0.001			<0.001
NIH	288 (19.2)	207 (15.9)	81 (40.3)		21 (7.4)	186 (18.3)	
Other	1212 (80.8)	1092 (84.1)	120 (59.7)		261 (92.5)	831 (81.7)	
Study condition according to ICD 10				<0.001			0.03
Infections	69 (4.6)	64 (4.9)	5 (2.5)		22 (7.8)	42 (4.1)	
Oncology	608 (40.5)	474 (36.5)	134 (66.7)		73 (25.9)	401 (39.4)	
Haematology	23 (1.5)	19 (1.5)	4 (2.0)		3 (1.1)	16 (1.6)	
Endocrinologic diseases	85 (5.7)	79 (6.1)	6 (3.0)		21 (7.4)	58 (5.7)	
Psychiatric diseases	119 (7.9)	109 (8.4)	10 (4.9)		28 (9.9)	81 (8.0)	
Neurologic diseases	62 (4.1)	58 (4.6)	4 (2.0)		14 (5.0)	44 (4.3)	
Eye	12 (0.8)	2 (1.0)	10 (0.8)		2 (0.7)	8 (0.8)	
Ear	2 (0.1)	2 (0.1)	0 (0.0)		1 (0.3)	1 (0.1)	
Cardiovascular diseases	123 (8.2)	118 (9.1)	5 (2.5)		30 (10.6)	88 (8.6)	
Lung diseases	59 (3.9)	53 (4.1)	6 (3.0)		10 (3.5)	43 (4.2)	
Digestive diseases	43 (3.3)	4 (2.0)	47 (3.1)		15 (5.2)	28 (2.7)	
Skin	19 (1.3)	18 (1.4)	1 (0.5)		3 (1.1)	15 (1.5)	
Musculoskeletal disorders	51 (3.4)	48 (3.7)	3 (1.5)		12 (4.3)	36 (3.5)	
Urology	76 (5.1)	72 (5.5)	4 (2.0)		20 (7.1)	52 (5.1)	
Pregnancy	26 (1.7)	23 (1.8)	3 (1.5)		5 (1.8)	18 (1.8)	
Perinatal disorders	10 (0.7)	10 (0.8)	0 (0.0)		3 (1.1)	7 (0.7)	
Labour	2 (0.1)	2 (0.1)	0 (0.0)		1 (0.3)	1 (0.1)	
Trauma	39 (2.6)	35 (2.7)	4 (2.0)		6 (2.1)	29 (2.8)	
Extern	1 (0.1)	1 (0.1)	0 (0.0)		1 (0.3)	0 (0.0)	
Other	67 (4.5)	61 (4.7)	6 (3.0)		12 (4.3)	49 (4.8)	
Study intervention				<0.001			0.10
Drug	895 (59.7)	741 (57.0)	154 (76.6)		143 (50.7)	598 (58.8)	
Procedure	231 (15.4)	211 (16.2)	20 (9.9)		55 (19.5)	156 (15.3)	
Behaviour	112 (7.5)	107 (8.2)	5 (2.5)		32 (11.3)	75 (7.4)	
Device	64 (4.3)	62 (4.8)	2 (1.0)		14 (5.0)	48 (4.7)	
Vaccine	17 (1.1)	12 (0.9)	5 (2.5)		3 (1.1)	9 (0.9)	
Gene therapy	5 (0.3)	4 (0.3)	1 (0.5)		0 (0.0)	4 (0.4)	
Other/not applicable	176 (11.7)	162 (12.5)	14 (7.0)		35 (12.4)	127 (12.5)	
Study phase				<0.001			<0.01
Phase I	137 (9.1)	113 (8.7)	24 (11.9)		19 (6.7)	94 (9.2)	
Phase II	357 (23.8)	281 (21.6)	76 (37.8)		45 (16.0)	236 (23.2)	
Phase III	230 (15.3)	206 (15.9)	24 (11.9)		51 (8.1)	155 (15.2)	
Phase IV	177 (11.8)	168 (12.9)	9 (4.5)		48 (17.0)	120 (11.8)	
Phase I/II	72 (4.8)	56 (4.3)	16 (8.0)		6 (2.1)	50 (4.9)	
Phase II/III	43 (2.9)	35 (2.7)	8 (4.0)		6 (2.1)	29 (2.8)	
Not applicable	484 (32.3)	440 (33.9)	44 (21.9)		107 (37.9)	333 (32.7)	

* E-mail address of principal investigator

** Chi-squared test

Categories with less than six counts overall were added to category "Other" or "Other/not applicable" for the chi-square test.

Agreement to disclose study details was very high for most individual items (Additional file 1). Willingness to register study details was lowest for details of planned subgroup analyses (49% willingness to register), sample size calculation (57%), and planned analyses methods (58%). However, disclosing study documents, in particular the study protocol and financial agreements, was found to be most problematic with only 31% of respondents willing to disclose these publicly. 151 investigators (54%) would be willing to present results of their studies in a register. However, only 89 participants (32%) would be willing to present results before submission to a peer-reviewed journal even if journal editors would accept such a presentation (a situation that would be comparable to today's practice regarding presentation at conferences).

**Figure 2:**
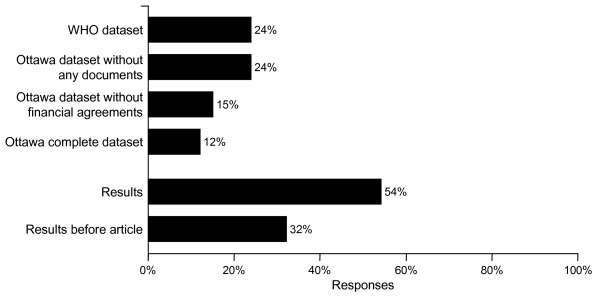
**Willingness to disclose study details**. For description of each data set see Table 1.

Sixty-eight responders (24%) were willing to disclose all items required by the WHO – and clinicaltrials.gov. Only 34/282 (12%) agreed to disclose all details proposed by the Ottawa Group (Figure 2).

If disclosing documents would not be required, 67/282 (24%) participants would be willing to disclose all other details. Not requiring registration of planned analyses methods including subgroup analyses only slightly increased this proportion (70/282; 25%). Restricting the data set to study details not identified as sensitive for competitive reasons by industry (scientific title, intervention, sample size calculation, outcome measures) also increased this proportion only slightly (71/282; 25%). There was no association between study phase and willingness to register details according to the data set proposed by the Ottawa Group (test for trend p = 0.59). Logistic regression indicated no association between characteristics of non-responders (study sponsored by the National Institutes of Health and phase II study in oncology) and willingness to disclose details (p > 0.15 for each explanatory variable).

## Discussion

This is the first survey on opinions of academic researchers suggesting that principal investigators of non-industry sponsored studies are reluctant to disclose all data items proposed by the Ottawa Group. Disclosing the study protocol and financial agreements were found to be most problematic.

As with any survey, our study is susceptible to non-response bias. The response rate was low with only 22% of potential participants responding. It is well known that web-based surveys have lower response rates than postal surveys [8]. Exact reasons for this low response rate remain unclear for us but might be related to the length of the questionnaire, the absence of any incentive, or the topic itself [9]. Nevertheless, we found no association between characteristics associated with non-response and the willingness to disclose study details. Although the impact of non-response seems therefore less problematic this study should be viewed as exploratory. Socially desirable responses might be another problem often encountered in surveys. Drawing conclusions about the actual habit of respondents is therefore often problematic [10]. Given the recent debate about trial registration and the requirement of editors of major journals to register a clinical study, one might expect that respondents would favour registration simply to conform with opinion leaders. The high proportion of respondents willing to disclose individual study details might be viewed as an indication for socially desired response habit. However, willingness to disclose all proposed items was low indicating that respondents differentiated well between different items. Finally, because of limited information available, we were not able to explore characteristics of investigators willing to disclose study details such as country of origin or host institution.

Recent debates about the WHO Trial Registration Data Set have focused on the reluctance of industry to disclose particular study details [11,12]. Representatives of industry argue that some study details are sensitive for competitive reasons and that these details might be disclosed with some delay [13]. However, academic research is also competitive nowadays and researchers might therefore also be reluctant to disclose study details. This reluctance might be even more pronounced for study registers adopting the data set proposed by the Ottawa Group [6]. The main reasons for prospective trial registration correspond to publication and outcome reporting bias [14,15]. For example, trial participants are at risk of being misled when their consent and the trial design are not fully informed by prior research or institutional review board (IRB) members cannot fully weigh risks and benefits when some unknown proportion of the relevant data is unavailable for review [16]. Consequently, it is an ethical obligation of researchers to publish all relevant details of their clinical studies and publicly available clinical study registers help to fulfil this obligation [17].

## Conclusion

As our survey suggests, academic researchers might be reluctant to disclose details of their study. Future discussions on trial registration should not only focus on industry but also on academic researchers. Consequences of more detailed registration of studies should be openly discussed when considering a minimum data set for a given trial register. These potential consequences not only include issues related to commercial and academic competition but also the increasing administrative workload of clinical researchers. Lastly, it seems important to disseminate the ethical obligation to register clinical studies more broadly.

## Competing interests

ST was a member of the Ottawa Group. MS declares that he has no competing interests.

## Authors' contributions

MS and ST equally participated in study design, statistical analysis and writing of the manuscript. Both authors had full access to all of the data in the study and take responsibility for the integrity of the data and the accuracy of the data analysis. Both authors read and approved the final manuscript.

## Pre-publication history

The pre-publication history for this paper can be accessed here:



## Supplementary Material

Additional file 1 All questions and corresponding results. The data provide represent the results of the survey. Click here for file
