# Supplementary material for: Opinions on registering trial details: a survey of academic researchers
Source: BMC Health Serv Res. 2008 Jan 23;8:18. doi: 10.1186/1472-6963-8-18 (PMC2245930; doi:10.1186/1472-6963-8-18)
Supplement: Additional file 1 — All questions and corresponding results. The data provide represent the results of the survey. [file 1472-6963-8-18-S1.doc]

| **Additional file 1: All questions and corresponding results** | | | | | |  |  |  |  |  |
| --- | --- | --- | --- | --- | --- | --- | --- | --- | --- | --- |
|  |  |  |  |  |  | n=282 | | | | |
|  | Variables | | | | | Yes | No | Don't know, can't decide | Don't want to answer. | Not applicable |
| Contacts and funding | | | |  |  |  |  |  |  |  |
|  | Would you be willing to disclose the funding source(s)? | | | | | 232 (82.3%) | 16 (5.7%) | 13 (4.6%) | 1 (0.4%) | 20 (7.1%) |
|  | Would you be willing to disclose the primary sponsor? | | | | | 233 (82.6%) | 12 (4.6%) | 11 (3.9 %) | 0 (0%) | 26 (9.2%) |
|  | Would you be willing to disclose the secondary sponsor(s)? | | | | | 194 (68.8%) | 14 (5.0%) | 12 (4.3%) | 0 (0%) | 62 (22.0%) |
|  | Would you be willing to disclose the coordinating/principal investigator? | | | | | 272 (96.5%) | 3 (1.1%) | 3 (1.1%) | 0 (0%) | 4 (1.4%) |
|  | Would you be willing to disclose the responsible contact person? | | | | | 266 (94.3%) | 8 (2.8%) | 6 (2.1%) | 0 (0%) | 2 (0.7%) |
|  | Would you be willing to disclose the coordinating center(s)? | | | | | 256 (90.8%) | 6 (2.1%) | 5 (1.8%) | 0 (0%) | 15 (5.3%) |
|  | Would you be willing to disclose the recruitment center locations? | | | | | 242 (85.8%) | 18 (6.3%) | 8 (2.8%) | 1 (0.3%) | 13 (4.6%) |
| Title | | | |  |  |  |  |  |  |  |
|  | Would you be willing to disclose the official scientific title? | | | | | 258 (91.5%) | 12 (4.3%) | 12 (4.3%) | 0 (0%) | 0 (0%) |
|  | Would you be willing to provide a lay title? | | | | | 248 (87.9%) | 15 (5.3%) | 10 (3.6%) | 0 (0%) | 9 (3.2%) |
|  | Would you be willing to disclose the acronym (if applicable)? | | | | | 201 (71.3%) | 16 (5.7%) | 5 (1.8%) | 0 (0%) | 60 (21.3%) |
|  | Would you be willing to disclose the trial website (if applicable)? | | | | | 161 (57.1%) | 17 (6.0%) | 13 (4.6%) | 0 (0%) | 91 (32.3%) |
|  | Would you be willing to provide a lay description? | | | | | 233 (82.6%) | 23 (8.2%) | 17 (6.0%) | 0 (0%) | 9 (3.2%) |
| Key dates | | | |  |  |  |  |  |  |  |
|  | Would you be willing to disclose the registration date? | | | | | 249 (88.3%) | 14 (5.0%) | 12 (4.3%) | 2 (0.7%) | 5 (1.8%) |
|  | Would you be willing to disclose the date of the ethical approval? | | | | | 237 (84.0%) | 22 (7.8%) | 18 (6.4%) | 0 (0%) | 5 (1.8%) |
|  | Would you be willing to disclose the date of the recruitment start? | | | | | 245 (86.9%) | 19 (6.7%) | 13 (4.6%) | 1 (0.4%) | 4 (1.4%) |
|  | Would you be willing to disclose the date of the recruitment end? | | | | | 222 (78.7%) | 23 (8.2%) | 20 (7.1%) | 1 (0.4%) | 16 (5.7) |
|  | Would you be willing to disclose the date of the end of follow-up? | | | | | 208 (73.8%) | 32 (11.4%) | 26 (9.2%) | 1 (0.4%) | 15 (5.3%) |
|  | Would you be willing to disclose the date when the trial stopped? | | | | | 226 (80.1%) | 20 (7.1%) | 17 (6.0%) | 2 (0.7%) | 17 (6.0%) |
|  | Would you be willing to disclose the date when the trial was extended and the relevant key dates? | | | | | 198 (70.2%) | 26 (12.8%) | 24 (8.5%) | 1 (0.4%) | 23 (8.2%) |
|  | Would you be willing to disclose the date when the primary analysis is complete? | | | | | 172 (61.0%) | 59 (20.9%) | 39 (13.8%) | 2 (0.7%) | 10 (3.6%) |
|  | Would you be willing to disclose recruitment status? | | | | | 189 (67.0%) | 63 (22.3%) | 28 (9.9%) | 0 (0%) | 2 (0.7%) |
| Ethical approval | | | |  |  |  |  |  |  |  |
|  | Would you be willing to disclose the name of the ethics board (REB/IRB) for the primary site in each country? | | | | | 244 (86.5%) | 15 (5.3%) | 15 (5.3%) | 0 (0%) | 8 (2.8%) |
|  | Would you be willing to disclose the REB trial approval number? | | | | | 210 (74.5%) | 26 (9.2%) | 33 (11.7%) | 1 (0.4%) | 12 (4.3%) |
| Background | | | |  |  |  |  |  |  |  |
|  | Would you be willing to disclose the rationale of the trial? | | | | | 240 (85.1%) | 22 (7.8%) | 17 (6.0%) | 2 (0.7%) | 1 (0.4%) |
|  | Would you be willing to disclose reference(s) to systematic review(s) justifying the trial? | | | | | 198 (70.2%) | 45 (16.0%) | 28 (9.9%) | 3 (1.1%) | 8 (2.8) |
|  | Would you be willing to disclose justification of experimental intervention(s) and comparator(s) in control groups (if applicable) (dosage, duration, frequency, etc.)? | | | | | 173 (61.4%) | 59 (20.9%) | 26 (9.2%) | 2 (0.7%) | 22 (7.8%) |
|  | Would you be willing to disclose the trial objectives? | | | | | 249 (88.3%) | 20 (7.1%) | 12 (4.3%) | 1 (0.4%) | 0 (0%) |
| Eligibility criteria | | | |  |  |  |  |  |  |  |
|  | Would you be willing to disclose all inclusion criteria? | | | | | 247 (87.6%) | 28 (9.9%) | 6 (2.1%) | 0 (0%) | 1 (0.4%) |
|  | Would you be willing to disclose all exclusion criteria? | | | | | 243 (86.2%) | 30 (10.6%) | 8 (2.8%) | 0 (0%) | 1 (0.4%) |
|  | Would you be willing to disclose the disease/condition (medical subject heading)? | | | | | 257 (91.1%) | 18 (6.4%) | 6 (2.1%) | 0 (0%) | 1 (0.4%) |
|  | | | |  |  |  |  |  |  |  |
|  | Variables | | | | | Yes | No | Don't know, can't decide | Don't want to answer. | Not applicable |
| Intervention | | | |  |  |  |  |  |  |  |
|  | Would you be willing to disclose details of the interventions by study groups if applicable (this includes the following information: meaningful intervention name, route of administration, dose, treatment duration; detailed description of the intervention in case of complex or surgical interventions; this description should be as detailed as in a scientific journal article/the study report)? | | | | | 168 (59.6%) | 58 (20.6%) | 28 (9.9%) | 2 (0.7%) | 26 (9.2%) |
| Outcome measures | | | |  |  |  |  |  |  |  |
|  | Would you be willing to disclose details of the primary outcome(s) (this includes the exact method of how outcome(s) are assessed and the timepoint(s))? | | | | | 196 (69.5%) | 52 (18.4%) | 28 (9.9%) | 1 (0.4%) | 5 (1.8%) |
| Design | | | |  |  |  |  |  |  |  |
|  | Would you be willing to disclose the trial phase (phase I, II, III, or IV) if relevant? | | | | | 232 (82.3%) | 13 (4.6%) | 10 (3.6%) | 0 (0%) | 27 (9.6%) |
|  | Is your study a controlled trial? | | | | | 177 (62.8%) | 105 (37.2%) | 0 (0%) | 0 (0%) | 0 (0%) |
|  |  | In case of YES would you be willing to disclose the following information about the study design in a publicly available trial register: | | | |  |  |  |  |  |
|  |  | | Would you be willing to disclose the study design (e.g. parallel group, crossover, cluster, factorial)?* | | | 173 (83.2%) | 10 (4.8%) | 8 (3.9%) | 0 (0%) | 17 (8.2%) |
|  |  |  | Would you be willing to disclose the number of arms?* | | | 168 (81.6%) | 11 (5.3%) | 8 (3.9%) | 0 (0%) | 19 (9.2%) |
|  |  |  | Would you be willing to disclose the generation of the allocation sequence (by date of birth, computer generated random numbers, etc.)?* | | | 139 (67.2%) | 30 (14.5%) | 16 (7.7%) | 1 (0.5%) | 21 (10.1%) |
|  |  | In case of a randomized-controlled trial | | | |  |  |  |  |  |
|  |  | | Would you be willing to disclose details of the randomization (stratification, block size, etc.)?* | | | 149 (71.0%) | 26 (12.4%) | 16 (7.6%) | 0 (0%) | 19 (9.1%) |
|  |  |  | Would you be willing to disclose details of the allocation concealment?* | | | 144 (69.2%) | 28 (13.5%) | 14 (6.7%) | 0 (0%) | 22 (10.6%) |
|  | Is your study a masked / blinded trial? | | |  |  | 97 (37.5%) | 162 (62.6%) | 0 (0%) | 0 (0%) | 0 (0%) |
|  |  | In case of blinded / masked trial: | | | |  | | | | |
|  |  | | Would you be willing to disclose details about blinding (this includes an explicit statement about whether interventions were indistinguishable, whether/what tests for successful blinding are done, etc.)?* | | | 73 (47.4%) | 25 (16.2%) | 12 (7.8%) | 0 (0%) | 44 (28.6%) |
|  | Would you be willing to disclose other design features? * | | | | | 153 (55.4%) | 51 (18.5%) | 59 (21.4% | 4 (1.5%) | 9 (3.3%) |
|  | Would you be willing to disclose the framework of the study (e.g. superiority,  non-inferiority, equivalence trial)? | | | | | 169 (59.9%) | 39 (13.8%) | 43 (15.3%) | 2 (0.7%) | 29 (10.3%) |
|  | Would you be willing to disclose the target sample size? | | | | | 235 (83.3%) | 27 (9.6%) | 15 (5.3%) | 0 (0%) | 5 (1.8%) |
|  | Would you be willing to disclose details of the sample size calculation (this includes justifications for all underlying assumptions e.g. assumed events rates, minimally clinically meaningful differences, etc.)? | | | | | 155 (55.0%) | 67 (23.8%) | 47 (16.7%) | 1 (0.4%) | 12 (4.3%) |
|  | Would you be willing to disclose details of planned subgroup analyses? | | | | | 125 (44.3%) | 74 (26.2%) | 53 (18.8%) | 3 (1.1%) | 27 (9.6%) |
|  | Would you be willing to disclose details about the planned analyses methods (e.g. analyses will be based on all included patients, patients will be analysed in the group to which they were randomized regardless of the treatment received, patients will be analysed according to the received treatment)? | | | | | 157 (55.7%) | 66 (23.4%) | 45 (16.0%) | 2 (0.7%) | 12 (4.3%) |
| Documents | | | |  |  |  |  |  |  |  |
|  | Would you be willing to disclose consent forms (pdf-documents)? | | | | | 142 (50.4%) | 96 (34.0%) | 38 (13.5%) | 1 (0.4%) | 5 (1.8%) |
|  | Would you be willing to disclose the full protocol (pdf-document)? | | | | | 87 (30.9%) | 139 (49.3%) | 49 (17.4%) | 3 (1.1%) | 4 (1.4%) |
|  | Would you be willing to disclose contracts and financial arrangements? | | | | | 81 (28.7%) | 125 (44.3%) | 49 (17.4%) | 3 (1.1%) | 24 (8.5%) |
| Results | | | |  |  |  |  |  |  |  |
|  | Would you be willing to disclose results of the trials (includes details about the statistical methods used and all outcomes analysed including stratified/subgroup analyses; results should be presented in a format that allows other investigators to perform meta-analyses with the data)? | | | | | 151 (53.6%) | 67 (23.8%) | 59 (20.9%) | 2 (0.7%) | 3 (1.1%) |
|  | Assuming that journal editors accept publication of results in a register before the submission of a manuscript on the study results (a situation comparable to presentations at conference), would you be willing to disclose the results in a register before submission of the manuscript? | | | | | 89 (31.6%) | 119 (42.2%) | 68 (24.1%) | 1 (0.4%) | 5 (1.8%) |

* Because of computational problems, responding to these questions was not required to proceed with the questionnaire. The number of respondents is therefore lower compared to the other questions.
